# Towards a chemo‐free approach for follicular lymphoma

**DOI:** 10.1111/bjh.70126

**Published:** 2025-08-30

**Authors:** Stefano Luminari, Emiliano Barbieri, Maria Elena Nizzoli

**Affiliations:** ^1^ Division of Hematology Azienda Unità Sanitaria Locale ‐ IRCCS Reggio Emilia Italy; ^2^ CHIMOMO Department University of Modena and Reggio Emilia Reggio Emilia Italy; ^3^ Clinical and Experimental Medicine Doctorate School University of Modena and Reggio Emilia Modena Italy

**Keywords:** chemo‐free therapies, follicular lymphoma, immunotherapy

## Abstract

The therapeutic landscape of follicular lymphoma (FL) is undergoing a transformative shift driven by the advent of novel chemo‐free strategies that challenge the traditional chemo‐oriented paradigms; this shift offers promising alternatives for both newly diagnosed and relapsed or refractory (RR) patients. Available data support a full chemo‐free approach starting from second‐line therapy, with rituximab–lenalidomide (R2) or tafasitamab‐R2, whereas bispecific antibodies (bsAbs), Bruton's tyrosine kinase (BTK) inhibitors and chimeric antigen receptor (CAR) T‐cell therapies are available options after second relapse. In the near future, bsAbs, mainly in combination with lenalidomide, will likely be employed as first‐ or second‐line therapy, potentially fully replacing immunochemotherapy, whereas CAR T‐cell therapy will play a role in selected high‐risk patients. Given the different toxicity profiles of chemo‐free options, refined prognostic scores are awaited so as to properly allocate patients to the most appropriate therapy with the best trade‐off between efficacy and safety.

## INTRODUCTION

Follicular lymphoma (FL) is the most common indolent lymphoma.^1^ The majority of patients (85%–90%) present advanced stage disease and require systemic therapies when symptomatic. Although characterized by an indolent clinical course and excellent response to treatment, with most patients surviving for decades, there is the continuous risk of disease recurrence, and FL is still considered incurable. The clinical course of the disease is very heterogeneous: patients can either experience long remissions between therapies or have refractory disease or frequent relapses that significantly impact overall survival (OS). Indeed, while one‐third of patients are expected to receive only one line of therapy in their lifetime,^2^ up to 20% of patients face a dismal prognosis after first‐line therapy due to rapidly relapsing or resistant FL, which can reduce life expectancy to less than 5 years from diagnosis.^3^, ^4^ This is more common among patients who relapse within 2 years since first therapy (POD24) and is frequently linked to histological transformation (HT) into aggressive lymphoma.^5^, ^6^, ^7^ A similar reduction in patients' outcome is reported for patients who experience repeated relapses, with a median OS from their second relapse of 67.6 months.^8^

In light of these heterogeneous outcomes, the main goals of FL therapy today are to try to achieve the best control of the disease to reverse the adverse prognosis of a significant proportion of high‐risk patients and to try to avoid the risk of long‐term complications, mainly infections and second malignancies, which may impair the survival of the low‐risk FL population.^9^ So far, existing prognostic models such as Follicular Lymphoma International Prognostic Index (FLIPI), FLIPI2 and PRIMA‐PI are unable to accurately predict patients' prognosis at diagnosis, thus hindering treatment tailoring based on risk stratification.^10^

For many years, the use of chemotherapy, mainly anthracycline‐ or bendamustine‐based, has represented the main pillar of FL treatment. Today, it is recommended as initial therapy in combination with anti‐CD20 monoclonal antibodies (mAb) for high tumour burden, symptomatic patients.^11^ The use of immunochemotherapy (ICT) as first line provides excellent control of the disease, with approximately 80% of patients achieving complete response and a median progression‐free survival (PFS) estimated to be more than 8 years,^12^ while a small number of patients show early relapse. The choice of chemotherapy may impact HT risk since it has been suggested that anthracycline reduces the risk of HT compared to other therapies.^13^ So far, the actual risk of HT among novel chemo‐free options has not been extensively analysed.

ICT has also been identified as the preferred approach for patients experiencing relapse or refractory (RR) disease. In these cases, patients are usually retreated with alternative, non‐cross‐resistant combinations or, in selected cases, with intensified protocols such as autologous stem cell transplant (ASCT).^11^ The efficacy of ICT as second‐line (2L) therapy has been confirmed, with reduced response rates compared to first line and with median PFS of around 2 years.^14^ ASCT consolidation in patients achieving complete or partial response after first salvage is recommended by recently published guidelines^15^ and has been traditionally mainly considered in POD24 patients. Allogeneic stem cell transplantation (alloSCT) currently represents the only curative option in FL, but it has always played a minor role in FL therapy due to the high transplant‐related mortality and morbidity. Its use is usually restricted to very young and fit patients achieving second or subsequent remission and lacking other therapeutic options.

Notwithstanding the high efficacy of ICT in FL, concerns about its real benefit have been raised for several reasons: first, none of the currently available studies has shown that any single treatment modality was able to modify patient's overall risk of death. This was true in the FOLL05 trial, which compared several ICT regimens,^16^ in the Gallium trial, which compared the more potent obinutuzumab with rituximab,^17^ in the PRIMA trial, when rituximab maintenance was compared with observation in patients responding to first‐line therapy,^18^ as well as in the RELEVANCE trial, when the chemo‐free combination R2 was compared to ICT for the first‐line therapy.^19^ Second, during both treatment phase and follow‐up, ICT has safety issues that tend to accumulate with retreatments and represent a major limitation of this approach. Third, many novel non‐chemotherapy agents have been tested in FL, showing high efficacy mainly in the setting of RR cases. These agents include novel naked or conjugated mAb, immunomodulating agents, epigenetic modifiers, Bruton's tyrosine kinase (BTK) inhibitors, bispecific antibodies (bsAbs) and chimeric antigen receptor (CAR) T‐cell therapy. Although long‐term efficacy and safety data are still awaited, these chemo‐free options may challenge the conventional ICT approach to the disease, and the administration of these new agents, guided by refined prognostic scores, may ultimately fulfil the need to reach durable disease control, thereby minimizing the risk of long‐term toxicities.

**FIGURE 1:**
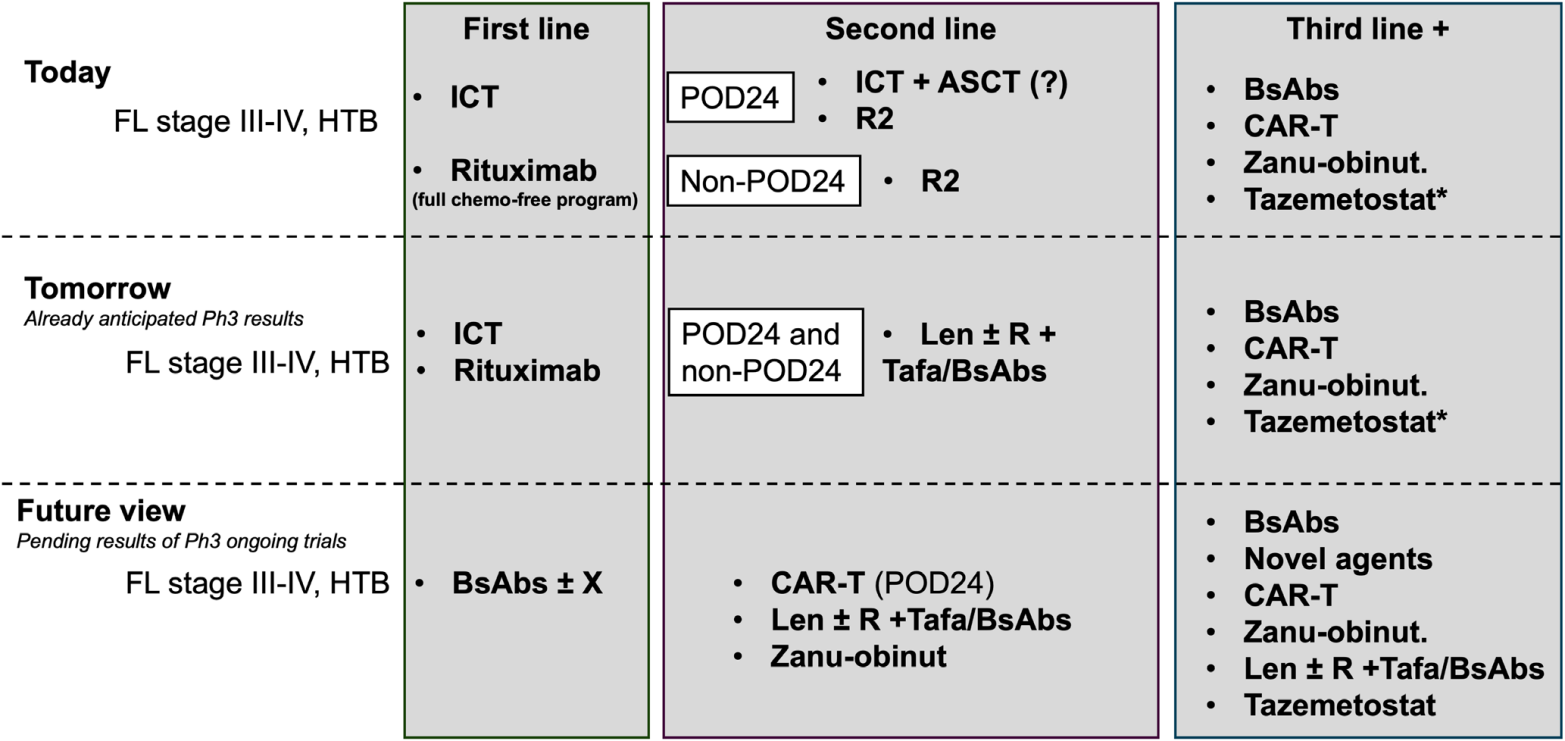
Evolving paradigms in FL therapy. ASCT, autologous stem cell transplant; bsAbs, bispecific antibodies; FL, follicular lymphoma; HTB, high tumour burden; ICT, immunochemotherapy; Len, lenalidomide; Ph3, phase 3 trials; R2, rituximab‐lenalidomide; Tafa, tafasitamab; Zanu‐obinut, zanubrutinib obinutuzumab. *Only FDA approved.

This review explores chemo‐free therapies in FL, starting from the first line and moving to second and later lines of therapy. Their potential to reshape treatment paradigms (Figure 1) and to ultimately improve patient outcomes is emphasized.

## CHEMO‐FREE THERAPY IN NEWLY DIAGNOSED PATIENTS

In the early 2000s, anti‐CD20 rituximab was the first cancer immunotherapy to be approved. Beyond its widely recognized role in combination with chemotherapy for the first‐line therapy of advanced stage symptomatic patients, rituximab has also been assessed as a single‐agent therapy in both low and high tumour burden patients. In a randomized study for low tumour burden patients, Ardeshna et al. compared rituximab monotherapy with observation; their results showed rituximab's ability to achieve a prolonged progression‐free interval, to delay the need for 2L therapies and to improve quality of life with minimal toxicity.^20^, ^21^ The use of single‐agent rituximab has also been tested in high tumour burden patients. In the long‐term follow‐up analysis of the first study to evaluate rituximab in the front‐line setting, 45% of treated patients achieved remission, maintained after 8 years of observation.^22^ The SAKK35‐03 study explored the duration of single‐agent rituximab treatment: no improvement in PFS was observed with prolonged duration of rituximab maintenance compared to a standard maintenance duration of 2 years.^23^ Consistent with the study by Martinelli et al., approximately one of three patients initially treated with single‐agent rituximab never required any additional therapy after a median observation time of 10 years. Similar results were reported in the 10‐year follow‐up update of two Nordic Lymphoma Group trials, which, however, reported a non‐negligible cumulative 20% incidence of transformed lymphomas.^24^ Therefore, rituximab monotherapy administered as induction and for up to 2 years of maintenance can be identified as the first chemo‐free approach to FL. Even if no direct comparison with ICT has been conducted, lower efficacy in terms of response and PFS rates versus more intensive treatment has been reported with this approach. Moreover, outcome data and transformation rate should be interpreted with caution since some degree of patient selection may have occurred. Nevertheless, a meaningful clinical benefit has been seen in a significant proportion of patients, suggesting that single‐agent rituximab may represent a good option, at least for frail or older patients. Bearing in mind the reduced efficacy versus standard ICT regimens, single‐agent rituximab might also be considered for selected patients unwilling to receive cytotoxic agents. In this latter scenario, rituximab might also be considered as a safe transition to the more effective chemo‐free regimens available for second or later lines of therapy.

Attempts have been made to test the efficacy of other chemo‐free options in the front‐line setting. Unlike rituximab, no other monoclonal antibody has been investigated as a single agent for the initial treatment of patients with FL. More Convincing data have been reported for the combination of rituximab and lenalidomide (R2), which was compared to R‐chemo in the first‐line setting in the randomized phase II RELEVANCE trial; this study showed similar 6‐year PFS (60% vs. 59%) and OS rates (89% for both arms).^25^ Although R‐chemo showed slightly higher overall response rates (ORR) (65% vs. 61%) and complete response (CR) rates (53% vs. 48%), these differences were not statistically significant and did not impact long‐term outcomes or HT rates. Exploratory analyses confirmed R2 efficacy across subgroups regardless of disease stage, FLIPI score or tumour bulk. Unfortunately, having failed the superiority hypothesis with which the trial was designed, the results of the RELEVANCE trial could not be used to approve and thus to reimburse the use of R2 as a first‐line chemo‐free regimen in most countries. The RELEVANCE trial supports R2 as a durable, effective, chemo‐free option for advanced FL, minimizing toxicity without sacrificing efficacy, which makes it ideal for older or comorbid patients. Based on these data, some countries introduced R2 among viable options for first‐line therapy, whereas in remaining areas, R2 is increasingly being adopted as standard 2L therapy.

## CHEMO‐FREE OPTIONS IN 2L THERAPY OF FL

Table 1 summarizes currently available chemo‐free options in RR FL. Single‐agent rituximab and R2 currently represent the only two chemo‐free options approved by the European Medicines Agency (EMA) for patients RR after one prior line of therapy. R2 obtained first approval in RR FL thanks to the phase III AUGMENT trial, which compared R2 to R‐placebo; the trial found an ORR of 78%, with a 34% CR rate and median PFS of 27.6 months.^26^, ^27^ These efficacy data were confirmed in a second trial (MAGNIFY), which evaluated a longer treatment schedule including R2 maintenance.^28^ The combination between lenalidomide and obinutuzumab is feasible,^29^ but efficacy data compared to R2 are lacking.

**TABLE 1:** Currently approved chemo‐free regimens in relapsed/refractory follicular lymphoma.

Agent	Approved by	Line	Trial, phase	N. of patients	Response rate	Median follow‐up (months)	mPFS (months)	mDOR (months)	mOS (months)
Rituximab‐lenalidomide	FDA, EMA	2L+	AUGMENT, phase 3	358	ORR 78%, CR 34%	65.9	27.6	NA	NR
Tafa‐R2	FDA	2L+	inMIND, phase 3	548	ORR 83.5%, CR 49.4%	14.1	22.4	21.2	NR
Zanubrutinib‐obinutuzumab	FDA, EMA	3L+	ROSEWOOD, phase 2	217	ORR 69%, CR 39%	20.2	28	NR	NR
Tazemetostat	FDA	3L+	NCT01897571, phase 2	45 EZH2^mut^ 54 EZH2^wt^	ORR 69%, CR13% ORR 35%, CR 4%	22 35.9	13.8 11.1	10.9 13	NR
Mosunetuzumab	FDA, EMA	3L+	GO29781, phase 2	90	ORR 80%, CR 60%	37	24	35.9	NR
Epcoritamab	FDA, EMA	3L+	EPCORE NHL‐1, phase 2	128	ORR 82%, CR 63%	27	15.4	NR	NR
Odronextamab	EMA	3L+	ELM‐2, phase 2	128	ORR 80%, CR 73%	20	20.7	22.6	NR
Axi‐cel	FDA, EMA	3L+ (4L+ EMA)	ZUMA‐5, phase 2	127	ORR 94%, CR 79%	41.7	40.2	38.6	NR
Tisa‐cel	FDA, EMA	3L+	ELARA, phase 2	97	ORR 86%, CR 68%	53	53.3	NA	NR
Liso‐cel	FDA, EMA	3L+	TRANSCEND FL, phase 2	130	ORR 97%, CR 94%	18.9	NR	NR	NR

Abbreviations: 2L+, second line or further; 3L+, third line or further; 4L+, fourth line or further; CR, complete response rate; mDOR, median duration of response; mOS, median overall survival; mPFS, median progression‐free survival; NA, not available; NR, not reached; ORR, overall response rate.

Recent real‐world data are consistent with those from the AUGMENT trial, thus reinforcing the R2 regimen's effectiveness even in a larger study population.^30^ Additionally, in this real‐world setting, bulky disease and rituximab refractoriness were associated with shorter PFS.

R2 has also been evaluated as maintenance after R‐chemo in older RR patients, showing a non‐significant clinical benefit and higher toxicity compared to rituximab maintenance.^31^

R2's manageability profile paved the way for combinations with other chemo‐free approaches. Recently published preliminary data of R2 in combination with tafasitamab (TafaR2) compared with R2 plus placebo highlighted a significant improvement in treatment efficacy, leading to Food and Drug Administration (FDA) approval after first‐line therapy. Tafasitamab is an anti‐CD19 mAb that mediates B‐cell lysis via apoptosis and the immune effector mechanisms antibody‐dependent cellular cytotoxicity and phagocytosis. Observed metabolic‐CR rate was 49.4% in the tafasitamab arm versus 39.8% with R2, and median PFS was 22.4 months versus 13.9 months, respectively, representing a 57% reduction in risk of progression, relapse or death.^32^ This trial enrolled 32% POD24 patients and 43% patients refractory to anti‐CD20 mAb; a PFS benefit was observed among all risk groups. The discontinuation rate due to adverse events (AEs) was slightly higher in the experimental arm, and OS data are awaited, but overall, tafasitamab‐R2 may represent a robust and durable chemo‐free treatment option for patients with 2L RR FL.

While assessing the role of available chemo‐free options in 2L RR FL, it must be acknowledged that none of these options has been directly compared with ICT, which has long been the standard approach to FL relapsing after one line of therapy. Several ongoing phase III trials have been designed to compare the efficacy of novel chemo‐free options with standard therapy, including the use of ICT, and they will hopefully fill this gap (Table 2). Analogously, the role of ASCT after salvage with R2 has not been determined and its role will be increasingly questioned with new immunotherapy‐based regimens becoming available in second line.

**TABLE 2:** Ongoing phase III trials comparing novel chemo‐free regimens with standard of care in 2L+.

Study	Drugs	Patient number	Population	Primary end‐point
NCT04712097 (CELESTIMO)	Mosunetuzumab‐lenalidomide versus R2	474	2L+ FL	PFS
NCT06149286 (OLYMPIA‐5)	Odronextamab‐lenalidomide versus R2	470	2L+ FL or MZL	PFS
NCT05409066 (EPCORE FL‐1)	Epcoritamab‐R2 versus R2	500	2L+ FL	PFS
NCT05371093 (ZUMA22)	Axi‐cel versus SOC	230	2L POD24 FL or 3L+ FL	PFS
NCT05888493 (LEDA)	Tisa‐cel versus R2 or RCHOP	108	3L+ FL	PFS
NCT05100862 (MAHOGANY)	Obinutuzumab‐zanubrutinib versus R2	750	2L+ FL or MZL	PFS

Abbreviations: 2L+ second line or further; 3L+ third line or further; CR, complete remission; FL, follicular lymphoma; GBenda, obinutuzumab‐bendamustine; MZL, marginal zone lymphoma; PFS, progression‐free survival; R2, rituximab‐lenalidomide; RCHOP, rituximab plus cyclophosphamide, doxorubicin, vincristine and prednisone; RCVP, rituximab plus cyclophosphamide, vincristine and prednisone; SOC, standard of care.

For the time being, even if an accurate assessment of the efficacy of chemo‐free regimens is not possible in this setting, the excellent safety profile and manageability of immunotherapies combined with their promising efficacy have nevertheless contributed to identifying the first relapse as when to start chemo‐free management in FL. In addition, the evidence that some ICT may impair the efficacy of subsequent therapies, especially in rapidly relapsing/progressing patients, will contribute to quickly reducing ICT use in the 2L setting.

For the same reasons, and with new agents available in third line, ASCT role in FL therapy is expected to be limited to regions where these drugs are not available.

## CHEMO‐FREE OPTION IN THIRD‐LINE RR FL AND BEYOND (3L+)

Several new chemo‐free options have been associated with meaningful activity in multiple relapsed FL. Most of these options have been specifically developed for 3L+ patients and have been added to previously described combinations or regimens (i.e. R2 or TafaR2) described above, but they can also be considered for more advanced lines of therapy if they have not been used before. Other chemo‐free options developed in the 3L+ setting include small molecule inhibitors, epigenetic regulators, new mAb, T‐cell engaging therapies and CAR T‐cell therapy.

B‐cell receptor (BCR) signalling inhibition has been pursued mainly through PI3K inhibitors (PI3Ki) and BTK inhibitors (BTKi). After raising initial interest and having obtained approval for RR FL, PI3Ki were largely withdrawn by regulatory agencies due to their toxicity profile. Single‐agent BTKi ibrutinib was initially evaluated in RR FL, with disappointing results,^33^ until next‐generation molecules emerged as more promising, especially as combination therapy. Indeed, second‐generation BTKi acalabrutinib demonstrated 75.9% ORR and 42.9% CR rates when combined with R2, but the frequency of serious AEs raised concerns that deserve further investigation in larger trials.^34^ In the ROSEWOOD trial, a different second‐generation BTKi, zanubrutinib, demonstrated promising results in combination with obinutuzumab in RR FL after ≥2 lines of therapy, with an ORR of 69% that outperformed obinutuzumab monotherapy (46%); the CR rate was 39% in the combination arm versus 19% in the monotherapy group.^35^ The combination also significantly extended PFS, with a median of 28.0 months compared to 10.4 months for obinutuzumab. These findings, together with zanubrutinib's manageable safety profile, led to FDA and EMA approval and warrant further development as combination therapies.

The knowledge of the overexpression of the anti‐apoptotic protein BCL‐2 in FL prompted the investigation of the BCL2 inhibitor venetoclax in this setting. Although However, early trials showed limited efficacy and high toxicity when combined with bendamustine,^36^ thus making the future of venetoclax in FL rather uncertain.

The pivotal role of mutations in epigenetic regulators such as *EZH2* in FL pathogenesis has prompted the development of target therapies. Tazemetostat, an oral *EZH2* inhibitor offering clinical benefits with a manageable safety profile, is emerging as a key therapy for RR FL. An earlier phase II trial showed significant benefits for *EZH2*‐mutant (mut) patients and activity in wild‐type *EZH2* (*EZH2wt*) patients, suggesting broader potential.^37^ A matched analysis confirmed robust efficacy, with ORRs of 71% for *EZH2mut* and 50% for *EZH2wt*, and similar PFS after adjusting for baseline differences (14.8 months for *EZH2mut* and 14.3 months for *EZH2wt*).^38^ Safety data highlighted the tolerability of tazemetostat, with manageable treatment‐related AEs like thrombocytopenia, neutropenia and anaemia, and no treatment‐related deaths. These findings led to FDA approval after two prior lines of therapy and enabled its development as combination therapy both in RR and in first‐line settings: tazemetostat‐R2, showing promising preliminary results,^39^ tazemetostat with bispecifics or tazemetostat chemotherapy.

Among new mAb, the CD19‐targeting antibody–drug conjugate loncastuximab showed preliminary activity in combination with rituximab in patients experiencing POD24 after first‐line therapy or relapsing after two or more therapies. A CR rate of 67% was observed in a phase 2 trial with no safety concerns.^40^ These early findings deserve further development.

T‐cell engagers consist of antibody‐based constructs designed to bridge immune effector cells to their target, thereby redirecting the immune response towards the tumour cells.

BsAbs are T‐cell engagers that represent a groundbreaking advancement in the treatment of FL and are rapidly gaining traction in clinical research. Clinical trials investigating anti‐CD3/CD20 bsAbs, including mosunetuzumab, epcoritamab and odronextamab, have shown promising efficacy in patients with RR FL as stand‐alone treatments, and ongoing trials are exploring their efficacy in combination regimens. A novel anti‐CD3/CD19 BsAb (AZD0486) is under development.^41^

Mosunetuzumab was the first bsAb to obtain both FDA and EMA approval for RR FL after two prior therapies. A phase I study demonstrated the feasibility and safety of intravenous fixed‐duration mosunetuzumab.^42^ A step‐up dosing schedule was preferred to optimize efficacy and to reduce cytokine release syndrome (CRS).

Mosunetuzumab approval was based on a phase II trial of 90 patients with a median of three prior therapies.^43^ Most were refractory to their last therapy (69%) or were double refractory (53%). Median time to first response was 1.4 months, and ORR and CR rates were 80% and 60%, respectively; 3‐year PFS was 43%. Common AEs included low‐grade CRS (44%); grade 3–4 AEs were rare: neutropenia (27%) and hypophosphatemia (17%). Serious AEs occurred in 47%, but only 4% discontinued due to AEs. A clinical benefit was observed in all subgroups. Long‐term data show a median duration of response (DOR) of 35.9 months and an OS of 82.4% at 36 months.^44^

Mosunetuzumab is currently being investigated as a subcutaneous fixed‐duration therapy in RR FL in combination with lenalidomide, zanubrutinib or polatuzumab.

In contrast to mosunetuzumab, epcoritamab and odronextamab have been developed as continuous therapies until progression or unacceptable toxicity.

At a median follow‐up of 17.4 months, the EPCORE NHL‐1 study demonstrated that epcoritamab was able to achieve an ORR of 82%, with 62.5% of patients achieving CR.^45^ Additionally, high rates of minimal residual disease (MRD) negativity were observed, which correlated with the not‐reached median PFS. Grade 1–2 CRS occurred in 65% of patients, while grade 3 CRS was reported in only 2%; the absence of grade 4 or 5 events indicated manageable toxicity. Slightly lower response rates were observed in patients with four or more previous lines of treatment, double refractory patients or those who were refractory to their last therapy. The fact that epcoritamab can be administered subcutaneously is a significant advantage as this reduces CRS risk through delayed and lower peak cytokine levels, thus potentially enhancing patient compliance and quality of life. These results enabled epcoritamab's development as combination therapy. Recent results from the phase I/II EPCORE NHL‐2 trial showed that epcoritamab–rituximab–lenalidomide achieved 98% ORR, with an 87% CR rate in RR FL regardless of risk stratification.^46^ Epcoritamab is FDA and EMA approved for RR FL after two prior therapies, and two phase III trials are currently comparing epcoritamab‐R2 versus R2 in the RR setting (NCT05409066) and epcoritamab‐R2 versus R2 versus ICT in the first‐line setting (NCT06191744). Moreover, phase II trials are evaluating the efficacy and safety of epcoritamab‐tazemetostat (NCT06575686) and epcoritamab‐R‐zanubrutinib (NCT06563596) in RR FL.

In the ELM‐2 trial, odronextamab demonstrated an ORR of 80%, with a CR rate of 73%.^47^ These robust efficacy outcomes are accompanied by durable remissions, with a median DOR of 22.6 months and a median PFS of 20.7 months. CRS risk was similar to other bsAbs, whereas a higher rate of infection (41% grade 3–5) was observed. As for epcoritamab, it must be taken into account that the ELM‐2 trial was heavily impacted by the COVID‐19 pandemic. ELM‐2 data resulted in EMA approval for RR FL and constitute the rationale for the ongoing OLYMPIA‐1 trial, comparing single‐agent odronextamab versus R‐chemo; the OLYMPIA‐2 trial, comparing odronextamab‐chemotherapy versus R‐chemotherapy for untreated FL; and the OLYMPIA‐5 trial, comparing odronextamab‐R2 versus R2 in RR FL.

In summary, mosunetuzumab, epcoritamab and odronextamab demonstrated comparable response rates without differences among risk categories and with a short median time to response. Follow‐up data are still limited, but suggest durable responses beyond 2 years. These products also displayed a similar toxicity profile, with CRS seen in approximately half of patients, but mainly of grade 1–2 and limited to the first cycle. Neutropenia and infections emerged as the main toxicity, with a higher number of fatal infections observed with epcoritamab and odronextamab, although mainly COVID‐19 related. Among the main differences between agents, route of administration, dosing of corticosteroid prophylaxis and treatment duration must be taken into account and may influence treatment selection. Subcutaneous administration currently available for epcoritamab may be preferred both by patients and infusion centres, whereas the dosing schedule might favour mosunetuzumab. Corticosteroid exposure within the first cycle is significantly higher for epcoritamab (1600 mg of prednisone) and odronextamab (180 mg of dexamethasone) compared to mosunetuzumab (60 mg of dexamethasone). Reduced steroid exposure together with a fixed‐duration administration may reduce the risk of infection, thus favouring mosunetuzumab. No real‐life experience regarding bsAbs in FL has been published so far. Longer follow‐up data of registrative trials and real‐world studies could shed precious light on long‐term efficacy and toxicity.

Anti‐CD19 CAR T‐cell therapy is another chemo‐free approach that has significantly improved treatment of lymphomas. Three CAR T‐cell constructs, axicabtagene ciloleucel (axi‐cel), tisagenlecleucel (tisa‐cel) and lisocabtagene maraleucel (liso‐cel), have shown remarkable activity in RR FL.

In the ZUMA‐5 trial, axi‐cel demonstrated an ORR of 94%, with a CR rate of 79% in patients with RR FL who had received multiple prior lines of therapy. CRS occurred in 78% of patients, with 6% experiencing severe CRS (grade ≥3) and 56% experiencing neurological events, including 15% with severe neurotoxicity (grade ≥3).^48^ Long‐term follow‐up data from ZUMA‐5 revealed a median DOR of 38.6 months, with a 36‐month OS rate of 75% and a median PFS of 40.2 months.^49^ The safety profile of axi‐cel includes manageable CRS and neurotoxicity, typically mitigated with supportive care such as tocilizumab and corticosteroids. Furthermore, recent data from the CIBMTR database confirmed similar efficacy and toxicity outcomes in a broader, real‐world cohort, thus reinforcing the clinical findings from ZUMA‐5.^50^

In the ELARA trial, tisa‐cel achieved a CR rate of 69% and an ORR of 86%.^51^ With a median follow‐up of 53 months, the estimated 45‐month PFS of the whole cohort was 52.9% (45% in POD24 patients, 52.9% in double‐refractory FL).^52^

Common AEs included CRS (49%, all grade 1–2) and neurological events (4%, with 1% grade ≥3), which were manageable.

The TRANSCEND‐FL study, the largest evaluation of CAR T‐cell therapy for RR FL, reported an ORR of 97% and a CR rate of 94% with liso‐cel in patients who had received multiple prior lines of therapy.^53^ These outcomes were consistent across subgroups, including those with high‐risk features and double‐refractory disease. Liso‐cel's safety profile was similar to that of tisa‐cel, with low rates of severe CRS (1%) and neurotoxicity (2%), and 13% of patients were managed in an outpatient setting. The median time to response was just 1 month, and efficacy remained robust, with no median PFS or DOR reached at a median follow‐up of 18.9 months.

Overall, phase II CAR T‐cell therapy trials have demonstrated efficacy, with a safety profile that seems to favour tisa‐cel and liso‐cel over axi‐cel. The longest follow‐up available describes a 45‐month PFS rate of 52.9%,^52^ and preliminary data suggest that lymphoma‐specific death seems uncommon during long‐term follow‐up.^54^ However, the curative potential of CAR T‐cell therapy in FL remains to be fully elucidated.

Ongoing phase III CAR T‐cell therapy trials (ZUMA‐22 for axi‐cel and LEDA for tisa‐cel) aim to compare this approach with the standard of care treatments R‐chemo and R2.

Currently, all three CAR‐T constructs have received FDA and EMA approval after two prior systemic therapies, except for axi‐cel which is EMA approved after three prior lines.

Although limited, real‐world data further corroborate the findings from clinical trials. The CIBMTR has reported an ORR of 84% and a CR rate of 84% for patients treated with axi‐cel, with toxicity rates similar to those observed in ZUMA‐5, including a higher incidence of severe neurotoxicity.^50^ The French DESCAR‐T registry has also reported favourable outcomes for tisa‐cel in 3L+ FL patients, with a CR of 88% and minimal severe CRS (1%) and neurotoxicity (4%).^55^

Overall, CAR T‐cell therapy currently represents the main alternative to bsAbs in RR FL after two prior lines. In contrast to bsAbs, which are ready‐to‐use drugs requiring multiple infusions mainly in an outpatient setting, CAR T is a one‐time therapy but has logistical challenges associated with time requirements for manufacturing, short‐term relocation to qualified medical centres and potential long‐term risks of infection and secondary cancers.^56^, ^57^

In contrast to large B‐cell lymphoma, where timing is crucial, long referral‐to‐infusion time will likely play a minor role in the therapeutic choice, that will mainly rely on the best balance between efficacy and toxicity. A direct comparison between bsAbs and CAR T would support this choice, but head‐to‐head trials are unlikely to be forthcoming. Therefore, a longer follow‐up of above‐mentioned trials and real‐world evidence is eagerly awaited in order to clarify curative potential and long‐term toxicities of these agents.

So far, the timing of bendamustine exposure might guide the choice between these two options since it is known that, in contrast to bsAbs, bendamustine can reduce the efficacy of CAR T‐cell therapy.^49^, ^58^ CAR T‐cell therapy may be preferred when HT is suspected, although not bioptically confirmed.^59^

Both therapies implicate high costs, with initial evidences that favour mosunetuzumab over CAR T.^60^, ^61^ Looking forward, ongoing trials (Table 3) will likely solve the CAR T/bsAbs dilemma by sequencing bsAbs as combination therapies in earlier lines and leaving CAR T‐cell therapy for selected patients in subsequent relapses. Finally, considering all available options and preliminary data on bsAbs retreatment,^62^ we do expect that alloSCT will be considered only in individual cases.

**TABLE 3:** Ongoing phase III trials comparing novel chemo‐free regimens with standard of care in first‐line setting.

Study	Drugs	Patient number	Population	Primary end‐point
NCT06337318	Mosunetuzumab versus rituximab	600	Low tumour burden stage 2–4 FL	PFS
NCT06284122 (MorningLyte)	Mosunetuzumab‐lenalidomide versus R/G‐CHOP/Benda	790	FLIPI 2–5 FL	PFS
NCT06191744	Epcoritamab‐R2 versus R2 versus R/G‐CHOP/Benda	900	Stage III–IV FL or bulky stage II	CR30
NCT06091254 (OLYMPIA‐1)	Odronextamab versus RCHOP/RBenda	473	Stage III–IV FL or bulky stage II	DLT, TEAE,CR30
NCT06097364 (OLYMPIA‐2)	Odronextamab‐CHOP/CVP +/− maintenance versus RCHOP	733	Stage III–IV FL or bulky stage II	DLT, TEAE,CR30
NCT06549595 (SOUNDTRACK‐F1)	AZD0486 + rituximab versus RCHOP/RCVP/RBenda	1015	Stage II–IV FL and FLIPI 2–5	TEAE, RP3D, PFS

Abbreviations: CR30, complete remission at 30 months; DLT, dose‐limiting toxicity; FL, follicular lymphoma; GBenda, obinutuzumab‐bendamustine; PFS, progression‐free survival; R2, rituximab‐lenalidomide; RBenda, rituximab‐bendamustine; RCHOP, rituximab plus cyclophosphamide, doxorubicin, vincristine and prednisone; RCVP, rituximab plus cyclophosphamide, vincristine and prednisone; RP3D, recommended phase III dose; TEAE, treatment emergent adverse event.

## CONCLUSION

The landscape of FL is undergoing a revolutionary transformation, driven by the advent of novel chemo‐free therapies that challenge conventional chemo‐oriented treatment paradigms. In this review, we describe chemo‐free options that can be considered starting from first‐line therapy on. While ICT is still the reasonable choice for first‐line therapy of FL, the available data already suggest a full chemo‐free approach starting from 2L RR FL. While a plethora of chemo‐free options are available from the second relapse on, very few options are currently available for 2L treatment. However, this scenario is likely to change very soon, when approval of tafa‐R2 is completed as well as when results from other ongoing phase III trials are revealed. In this setting, the use of bsAbs, mainly in combination with lenalidomide, will likely dominate 2L therapy, with a possible role for CAR T‐cell therapy in patients with rapidly progressing disease. However, as a significant proportion of patients ineligible for T‐cell engagers or CAR T will remain, the more conventional chemo‐free therapies (R2, tafa‐R2, obinutuzumab‐zanubrutinib) are excellent options in these cases.

Additional changes will be seen in the near future, when ongoing randomized trials for newly diagnosed patients are concluded: at least three large randomized studies are currently enrolling untreated FL patients to compare the efficacy of bsAbs, either as single agents or combined with lenalidomide or standard ICT (Table 3). The preliminary results of phase II studies investigating single‐agent mosunetuzumab or epcoritamab + R2 as front‐line therapy are very promising in terms of efficacy and safety data.^63^, ^64^ Caution, however, is always suggested before fully transitioning to a chemo‐free strategy based on novel agents. Although FL remains an incurable disease for most patients, they can still enjoy prolonged survival with excellent quality of life, already achievable with currently available old‐fashioned options until the changes anticipated by the chemo‐free revolution have been carefully investigated through real‐life studies and prolonged follow‐up. Moreover, reliable prognostic scores and validated minimal residual assessment should guide this transition to an individualized chemo‐free approach.

Continued innovation, collaboration and a commitment to equity are critical to realizing the full potential of these advancements, which may ultimately transform the outlook for patients with this historically incurable disease.

## AUTHOR CONTRIBUTIONS

All authors have contributed equally to this article.

## CONFLICT OF INTEREST STATEMENT

SL received honorarium for collaborations with advisory board for Roche, Beigene, Incyte, Abbvie, Kite, Novartis, BMS and is a speaker's bureau member for Roche, Abbvie, BMS, Incyte. The remaining authors declare no conflict of interest.
